# Exosomes, an Unmasked Culprit in Neurodegenerative Diseases

**DOI:** 10.3389/fnins.2017.00026

**Published:** 2017-01-31

**Authors:** Federico N. Soria, Olatz Pampliega, Mathieu Bourdenx, Wassilios G. Meissner, Erwan Bezard, Benjamin Dehay

**Affiliations:** ^1^Institut des Maladies Neurodégénératives, UMR 5293, Université de BordeauxBordeaux, France; ^2^Centre National de la Recherche Scientifique (CNRS), Institut des Maladies Neurodégénératives, UMR 5293Bordeaux, France

**Keywords:** exosomes, extracellular vesicles, neurodegeneration, cell-to-cell transmission, biomarkers

## Abstract

Exosomes are extracellular nanovesicles (30–100 nm) generated from endosomal membranes and known to be released by all cell lineages of the Central Nervous System (CNS). They constitute important vesicles for the secretion and transport of multilevel information, including signaling, toxic, and regulatory molecules. Initially thought to have a function merely in waste disposal, the involvement of exosomes in neuronal development, maintenance, and regeneration through its paracrine and endocrine signaling functions has drawn particular attention in recent years. These vesicles, being involved in the clearance and cell-to-cell spreading of toxic molecules, have been naturally implicated in aging, and in several neurodegenerative diseases associated with pathological conversion of proteins, as well as in the transport of other disease-associated molecules, such as nucleic acids or pro-inflammatory cytokines. Our understanding of such unique form of communication may provide not only answers about (patho)physiological processes in the brain, but can also offer means to exploit these vesicles as vehicles for the delivery of biologically relevant molecules or as tools to monitor brain diseases in a non-invasive way. A promising field in expansion, the study of exosomes and related extracellular vesicles has just commenced to unveil their potential as therapeutic tools for brain disorders as well as biomarkers of disease state.

## Introduction

Among intracellular organelles, eukaryotic cells also contain organelles that are released into the microenvironment. These membranous extracellular vesicles can be classified according to their size into exosomes, ectosomes, and apoptotic blebs (Mathivanan et al., 2010), many of which exhibit pleiotropic biological functions. In particular, exosomes were initially thought to act as cellular waste disposal compartments because of their extrusion from the cell, but it was only recently discovered that exosomes contain not only cellular proteins but also act as natural carriers of nucleic acid material in the form of DNA, mRNA transcripts, miRNA, and noncoding RNA (Schorey and Bhatnagar, 2008). They are released under normal as well as pathological conditions and since, have been recognized as a critical messenger for short and long-distance cell-to-cell communication. As such, cells use exosomes in the central nervous system (CNS) as a major route of secretion in order to get rid of waste membranes, harmful RNA or proteins or, more importantly, to act as messengers, and signal carriers to other neural cells, modifying their function in normal physiology as well as in states of disease (Zappulli et al., 2016). In this regard, given their ability to mediate intercellular communication, exosomes are increasingly being considered as one of the actors in transporting pathological misfolded proteins that are involved in neurodegenerative diseases and in disseminating disease into and within the brain. Nevertheless, since exosomes would be capable of transporting drugs across the blood brain barrier, their therapeutic potential has generated great interest in the field.

## Exosomes and intercellular communication

Studies in a variety of model systems have provided insights about origin, composition, and functions of exosomes. The physiological role of these vesicles is to transport biomolecules between different cells, being considered as a mechanism of paracrine (and possibly endocrine) communication. However, most of the experimental investigations are related to pathophysiological conditions and disease models, from which the physiological function has been inferred. Therefore, it has been assumed that the function of exosomes is maintained during physiological and pathological conditions, although further basic studies about their role would be key to corroborate this assumption.

### Exosome biogenesis

Three main types of vesicles have been described so far: exosomes, ectosomes and apoptotic blebs. Exosomes range from 30 to 100 nm in diameter, whereas ectosomes range between 50 and 1000 nm and apoptotic blebs, released by dying cells, between 50 and 5000 nm. Among them, the best characterized ones are exosomes. Exosomes are membrane-bound spherical nanovesicles of endocytic origin that are released by almost all cell types, and which can be found in abundance in body fluids (Simpson et al., 2008). The biogenesis of exosomes begins with the invagination of endosomal membranes to form multivesicular bodies (MVBs) containing intraluminal vesicles (ILVs) that range from 30 to 100 nm in diameter (Simons and Raposo, 2009). MVBs can either fuse with lysosomes for cargo degradation, or with the plasma membrane, which leads to the release of the ILVs to the extracellular space as exosomes (Mathivanan et al., 2010).

### Exosome membrane markers

General markers for exosome include enrichment in CD9, CD63, CD81, and Heat Shock Cognate 70 (HSC70) proteins, as well as the embryonic stem cell marker Nanog (Sheller et al., 2016). As described in this study, a combination of different methods is used to accurately characterize the exosomes; electron microscopy for shape and morphology, particle sizing, western blot and flow cytometry for detection of specific markers and cargo contents, and UV absorbance for DNA quantification. Altogether, a high-quality isolation method for exosomes, followed by their characterization and identification, is mandatory to distinguish exosomes from other microvesicles.

### Exosome cargo markers

The characterization of exosomal cargo will vary depending on the cell type that releases the exosome, and although the diversity in the cargo profile suggests different mechanisms of selection, there is still no study about the molecular details nor whether it is a random event. The myriad of results obtained from the studies that focus on the characterization of exosome content are gathered in different databases such as Exocarta, EVpedia, or Vesiclepedia (Mathivanan and Simpson, 2009; Kalra et al., 2012; Mathivanan et al., 2012; Simpson et al., 2012; Kim et al., 2013). They provide information about the protein, lipid or acid nucleic content of the different types of exosomes. Despite the variety in isolation techniques and different cell types used in these studies, it seems that a significant number of the proteins found in exosomes come from the biogenesis process, as it happens for components of the endosomal sorting complex for transport (ESCRT) pathway, Rab proteins needed for the formation and release of vesicles (Rab27A, Rab11B, ARF6), or the tetraspanins CD9, CD63, and CD81.

Cargo loading into the exosomes can be achieved by different mechanisms, although protein binding to the plasma membrane and its subsequent oligomerization seems to be the main process for cargo acquisition. However, any protein that binds to an exosome cargo could potentially be loaded into the nascent exosomes, as it is the case for RNAs, which typically exist in complex with proteins. Therefore, the selective trafficking of these macromolecules is linked to the delivery of their binding partners. Alternatively, cargo proteins can be covalently linked to exosomes (Yang and Gould, 2013).

Lipids are also loaded into exosomes as cargoes, in which case they share many characteristics with the lipid membranes of their cells of origin. Their main differential characteristic is the enrichment in phosphatidylserine in the outer face of the exosomal membrane, which facilitates the internalization by recipient cells (Subra et al., 2007; Fitzner et al., 2011).

Regarding nucleic acids, exosomes are enriched in small RNAs as compared with DNA, although almost any type of RNA has been found in these vesicles (Abels and Breakefield, 2016). However, the profile of the RNA content in exosomes differs substantially from that of the total cell (Guduric-Fuchs et al., 2012; Jenjaroenpun et al., 2013; Yagi et al., 2016). Similarly, the analysis of different secretomes led to the conclusion that exosomes derived from mesenchymal stromal cells (MSCs) in the bone marrow enhance neurite growth, as opposed to microvesicles or total secretome. The differential profiles in exosomal cargo content found in this study suggest that selective mechanisms underlie the processes of cargo selection and/or reception, which are still unexplored, and could open the door to the development of new therapeutic tools (Lopez-Verrilli et al., 2016).

### Exosome release and transmission

Although poorly investigated, exosome release is thought to be affected by other cellular events, and it has been described that synaptic glutamatergic AMPA and NMDA receptors modulate exosome release in differentiated neurons (Lachenal et al., 2011). Indeed, neuronal depolarization and calcium influx events into the neuron after ionomycin treatment enhanced exosome release (Lachenal et al., 2011), suggesting that this is an event that can be modulated and therefore suitable to be developed as a therapeutic tool. Several other works have confirmed the role of exosomes in modulating synaptic activity, as for example the polarized delivery of exosomes at synapses (Mittelbrunn et al., 2015). Also, endocannabinoids, which are secreted through extracellular vesicles produced by microglia, are able to inhibit presynaptic transmission (Gabrielli et al., 2015). Exosomes can indeed act as novel mechanisms of trans-synaptic communication, in which synaptogenic factors such as the presynaptic protein Evi are released into the synaptic cleft *via* these vesicles (Korkut et al., 2009).

Another rising issue in exosome research is that most of the studies use scenarios where a certain biomolecule is overexpressed, due to technical requirements or to the specific pathology that is being studied. It has to be pointed out that overexpression techniques can lead to a forced transmission event that otherwise would not occur in physiological conditions. This concern, together with the fact that exosome release is seldom the primary route of cell-to-cell transmission, question whether exosomes are a complementary but not necessary mechanism of paracrine/endocrine communication.

Once delivered, exosomes are taken up by the recipient cell, in a process that involves fusion with the plasma membrane, and different mechanisms of endocytosis that depend on the cell type (Abels and Breakefield, 2016). For example, in the CNS, neurons internalize exosomes through clathrin-dependent endocytosis or phagocytosis (Feng et al., 2010; Fruhbeis et al., 2013), while microglia internalizes these membrane vesicles through macropinocytosis (Fitzner et al., 2011). The uptake may depend on the cellular state of the recipient cell, since surface ligands recognize receptors on the exosome. However, an important question that remains unanswered about extracellular vesicles is whether the recipient cell has any role in the selection of the receiving cargo, or if this only depends on the emissary cell. Other factors that modulate the uptake of exosomes is the presence of heparin sulfate proteoglycans (HSPGs) on the plasma membrane (Atai et al., 2013; Christianson et al., 2013), or the blockage of specific receptors on the plasma membrane, as it was described for SR-B1 receptor (Plebanek et al., 2015).

## Exosomes in neurodegenerative diseases

**Figure 1:**
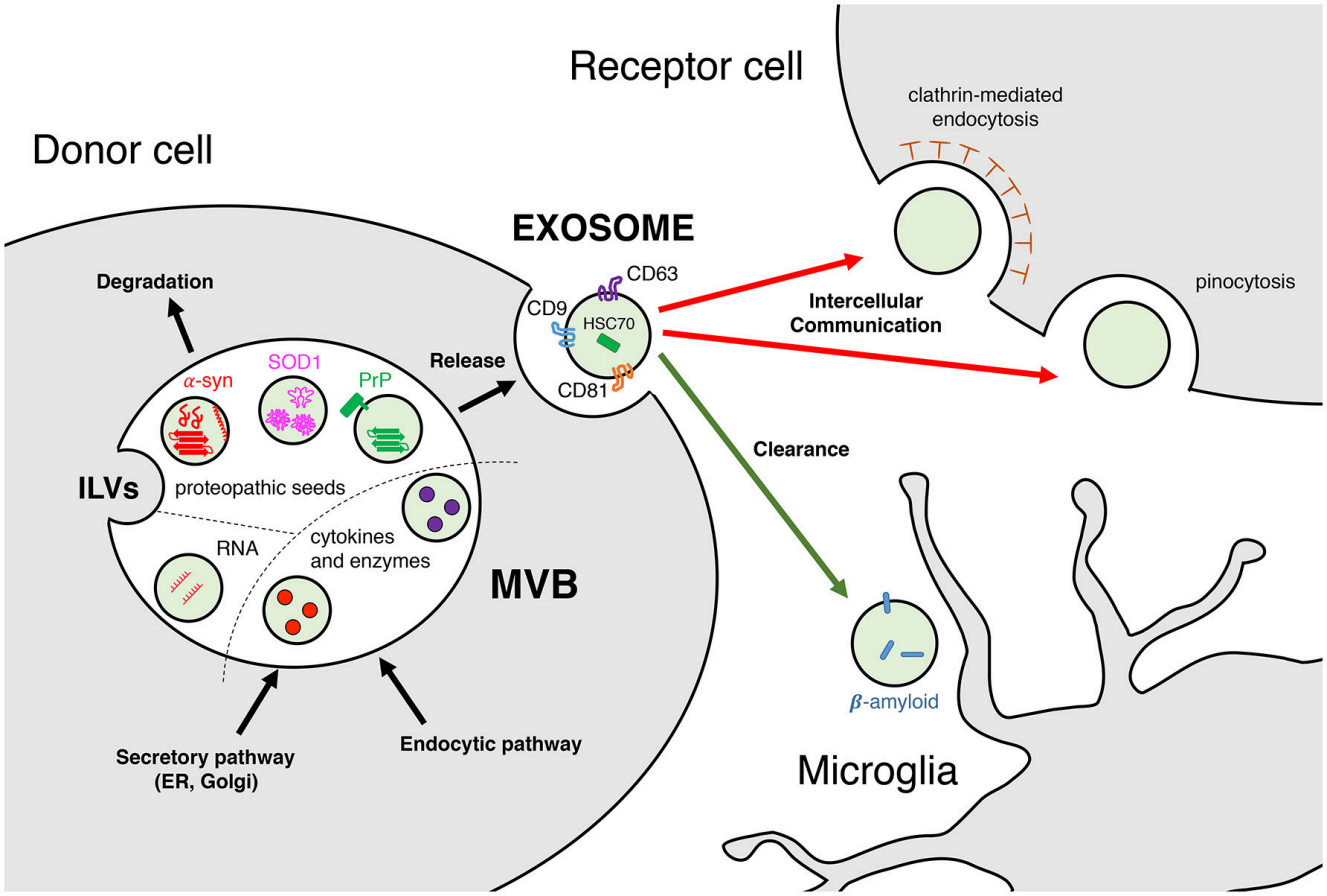
**Exosomes: biogenesis, composition, and function**. Exosomes are released upon fusion with the plasma membrane of multivesicular bodies (MVBs), which are constituted by intraluminal vesicles (ILVs) from the secretory (ER, Golgi) and/or endocytic pathways. Exosomal cargoes range from nucleic acids (mRNAs and miRNAs), cytokines and enzymes (IL-1β, proteases) to misfolded proteins (α-synuclein, SOD1, PrP) in their monomeric and/or oligomeric forms. These molecules can be carried either in the exosomal lumen or embedded in its membrane. Exosomes released to the extracellular space display a variety of markers, such as tetraspanins CD9, CD63, and CD81 or heat shock cognate 70. Intercellular communication is effectively achieved upon uptake by the receptor cell, either facilitated by clathrin-mediated endocytosis or by pinocytosis. Receptor cells can be in the nearby region (paracrine) or further beyond (endocrine). Clearance of toxic molecules contained within exosomes, such as β-amyloid, is thought to be mediated by microglia and macrophages.

Intercellular communication is a cornerstone in the research of neurodegenerative diseases. Beyond the complex molecular events that take place within the cell, the effects that these can have on neighboring or distant cells are a matter of intense investigation. This is mostly because communication often happens through the extracellular space, the compartment where it is easier to intervene, either by using a therapeutic tool to modify the disease, or by probing and sampling to diagnose or assess disease progression. During the last decade, among the several mechanisms for cell communication studied in neurodegenerative diseases, exosomes and extracellular vesicles have emerged as common players. Clearance and disposal of byproducts or toxic molecules, as well as cell-to-cell transmission of nucleic acids, cytokines and enzymes, but also aggregated or misfolded proteins, are among the several cargos being studied in brain pathophysiology (Figure 1). At this time, most of the advances have been achieved *in vitro*, but the results are vastly interesting, particularly for the prion-like spreading hypothesis applied in the understanding of neurodegenerative diseases.

### Prion diseases

Prions are infectious agents consisting of proteins misfolded into aberrant conformations that can transmit their misfolded state to “naïve” proteins of the same type and aggregate with themselves into oligomers and fibrils. According to the prion hypothesis (Prusiner, 1982), these infectious particles operate, thus, as proteopathic seeds that trigger a chain-reaction of refolding and self-aggregation, spreading the pathophysiological status to other cells, and ultimately causing neurodegeneration and loss of gray matter. Transmissible spongiform encephalopathies such as Creutzfeldt-Jacob disease in humans or scrapie in animals, are known to originate from this mechanism (Collinge, 2001). Similarly, in other neurodegenerative diseases such as Alzheimer's or Parkinson's Disease there is also a progressive accumulation of misfolded proteins such as β-amyloid, tau, and α-syn, among others, which occurs concomitantly with the spreading of the pathology between neuroanatomically connected regions. These characteristics have led to the hypothesis that these and other proteinopathies share a common originating mechanism, which has given rise to the “prion-like” hypothesis (Jucker and Walker, 2013).

The idea of exosome-mediated propagation in prion diseases was introduced in the last decade, when Fevrier et al. (2004) found that the prion protein (PrP)-expressing cells could release PrP^C^ (normal) and PrP^Sc^ (abnormal) in association with exosomes. The first report that linked *in vivo* the prion disease pathogenesis and exosomes came 4 years later, when Vella et al. (2008) found PrP^C^ associated to extracellular vesicles in the CSF of sheep. The interplay between prions and exosomes has just begun to be unveiled in recent years: The biogenesis of exosomes appears to be implicated both in the conversion of PrP^Sc^ (Yim et al., 2015) as well as in its packaging (Vella et al., 2007). Moreover, PrP^Sc^ can apparently alter the structure (Coleman et al., 2012) and cargo (Bellingham et al., 2012b) of exosomes, and upregulating the exosomal secretion pathway increase the infectiousness of PrP^Sc^ (Guo et al., 2016).

### Alzheimer's disease: β-amyloid and tau

Rajendran et al. (2006) first described *in vitro* the intracellular β-cleavage of the amyloid precursor protein (APP) in early endosomes, and the subsequent secretion of β-amyloid (Aβ) to the extracellular space via exosomes, suggesting a role of these vesicles in Aβ propagation. There have been no further reports associating exosomal secretion to Aβ spreading, but APP-C-terminal fragments have been found, indeed, in exosomes *in vitro* (Sharples et al., 2008) and *in vivo* (Perez-Gonzalez et al., 2012), which at least suggests a role of the exosomal secretory pathway in the generation of Aβ. Nonetheless, consensus within the field seems to be that direct cell-to-cell transmission is more prevalent than exosome-mediated transfer for the pathological spreading of Aβ (Bellingham et al., 2012b).

However, conflicting reports exist about the stabilization of Aβ toxic species inside MVBs. Some authors suggest that exosomes promote aggregation of Aβ, serving as nucleation hubs for plaque formation (Dinkins et al., 2014; Falker et al., 2016). This hypothesis would render exosomes as neuroprotective compartments, which clear toxic oligomeric species of Aβ in favor of the more stable and less toxic fibrils. In contrast, others propose that exosomes can indeed induce the disassembly of stable species into neurotoxic oligomers (Yuyama et al., 2012; Joshi et al., 2014), depending on whether the exosomes are of neuronal or myeloid origin, based on the idea that different membrane lipid compositions might affect Aβ assemblies.

Hyperphosphorylated misfolded tau, the primary component of neurofibrillary tangles and a cytopathological hallmark of AD, is associated to exosomes in the CSF of AD patients (Saman et al., 2012). Several *in vitro* studies where tau is overexpressed suggest that this microtubule-associated protein is secreted via exosomes (Chai et al., 2012; Simon et al., 2012), and transmitted trans-synaptically through the extracellular space (Lee et al., 2012; Liu et al., 2012), which proposes a mechanism for the progressive spreading of tauopathy in the brain of AD patients. Indeed, inhibition of exosomal secretory pathways reduces the propagation of Tau *in vitro* and *in vivo* (Asai et al., 2015) in a viral-mediated expression model. On the contrary, secretion of endogenous tau is regulated by neuronal activity (Pooler et al., 2013; Wu et al., 2016), and this physiological process appears to be exosome-independent (Pooler et al., 2013). Additional work in more intact preparations that avoid the possible artifacts from tau overexpression, is necessary to shed light into this subject.

The relevance of exosomes in proteinopathies should not only be explored from the point of view of intercellular transmission of proteopathic seeds. Indeed, there is still debate in the field about whether the pathological trait is transmitted by the misfolded protein itself, the so-called “prion-like” self-propagation, or other signals induce the aberrant folding in selectively vulnerable neurons (Walsh and Selkoe, 2016). Indeed, a recent study showed that misfolded seeds can be found all over the brain, still suggesting a propagation of proteopathic seeds, while only a subset of the brain will indeed display aggregation and neurodegeneration (Alibhai et al., 2016). Looking into the problem from the latter perspective, exosomes might be carriers of molecules such as nucleic acids or enzymes that could exert a pathophysiological modification in an endocrine manner. For instance, secretion of enzymes that degrade Aβ are associated to exosomes (Bulloj et al., 2010; Tamboli et al., 2010), and miRNAs transported within neuron-derived exosomes can activate glial cell functions, such as activation of microglial phagocytosis for the clearance of neurodegenerating dendrites (Bahrini et al., 2015). Furthermore, microglial and neuronal exosomes have been proposed to participate in the disposal of Aβ in the brain (Yuyama et al., 2012, 2015), a concept that becomes further relevant given that impaired Aβ clearance is a characteristic of AD (Mawuenyega et al., 2010). Altogether, the current state-of-the-art in the field shows that not only cell-to-cell transmission, but also exosome-mediated communication and clearance might be important players in AD.

### Parkinson's disease and other synucleinopathies

Alpha-synuclein (α-syn) is a major component of Lewy bodies (Spillantini et al., 1997), the neuropathological hallmark of Parkinson's Disease (PD) and Dementia with Lewy bodies (DLB). α-Syn is a pre-synaptic protein that exists in an equilibrium between monomeric and oligomeric states. Mutations and other disease-related factors affect the aggregation dynamics, favoring the balance toward fibrillization (Dehay et al., 2015). The neurodegenerative process observed in PD spreads through anatomically interconnected regions in the CNS (Braak et al., 2003), and the pathogenic trait of misfolded α-syn is thought to propagate through a prion-like process (Olanow and Prusiner, 2009; Lee et al., 2010). Very recently, a team demonstrated that exosomal α-syn species isolated from the CSF of PD and DLB patients is able to promote aggregation of α-syn (Stuendl et al., 2016). However, whether exosomes play a role in α-syn spreading is still a matter of debate.

Among all proteopathic seeds, most reports of secretion associated to exosomes and related vesicles belongs to the α-syn field (Emmanouilidou et al., 2010; Alvarez-Erviti et al., 2011a; Danzer et al., 2012; Kong et al., 2014; Shi et al., 2014; Tsunemi et al., 2014). The first report that identified α-syn showed that this is a protein present in synaptic vesicles. Due to its cellular function, a link to exosomes might be related to its intrinsic function within the exocytic machinery at the pre-synaptic terminal (Bendor et al., 2013). Despite the fact that the physiological role of α-syn is not completely understood, it has been suggested that α-syn is involved in synaptic vesicular processing (Vargas et al., 2014), where it binds to the membrane and undergoes conformational changes that facilitate the generation of the vesicle curvature (Varkey et al., 2010; Westphal and Chandra, 2013). It has also been reported that α-syn prefers membranes with high curvature (Jensen et al., 2011), like the ones found in the 50–100 nm exosomes. It is worth to note that non-monomeric, i.e., aggregated α-syn, is unable to perform this task, and it is not clear whether membrane association induces or prevents oligomerization (Bendor et al., 2013). In the first study that characterized the secretion of α-syn by unconventional secretory pathways, Lee et al. (2005) already discussed that its compartmentalization into vesicles could promote the misfolding of α-syn. A recent study demonstrated that exosomes accelerate α-syn aggregation (Grey et al., 2015), and that the lipid composition of the vesicles is critical for this process. The mechanism by which α-syn is incorporated into exosomes is still unknown, but given the vast number of genes linked to PD that have a role in endocytic and autophagy pathways such as *PARK2, GBA, LRRK2*, or *ATP13A2* (Gan-Or et al., 2015), the rapid advancement of research in vesicle trafficking might give some clues in the next years.

In multiple system atrophy (MSA), oligodendrocytes forming α-syn-containing aggregates named glial cytoplasmic inclusions (GCIs) accumulate α-syn, which is accompanied by demyelination and extensive dopaminergic neuronal loss throughout the brain. Although transgenic MSA models overexpressing α-syn under different oligodendrocyte-specific promoters have been studied for a decade (Fernagut and Tison, 2012), the link between oligodendroglial α-syn aggregation and neuronal death remains elusive. Moreover, as α-syn is a predominantly neuronal protein, it is also unknown if oligodendrocytes in a disease state can synthesize α-syn *de novo*, or if α-syn is readily transferred from neurons. In this regard, there are several reports indicating that oligodendrocytes and neurons communicate via exosomes, which supports the hypothesis of α-syn transfer between these two cell types (Kramer-Albers et al., 2007; Bakhti et al., 2011). This exosomal communication between neurons and oligodendrocytes includes the transfer of genetic information by miRNAs (Fruhbeis et al., 2013), which in fact are altered in MSA (Ubhi et al., 2014; Schafferer et al., 2016). Therefore, a potential involvement of the exosomal secretory pathway in the pathogenesis of MSA should not be overlooked.

### Amyotrophic lateral sclerosis and huntington's disease

Amyotrophic lateral sclerosis (ALS) is characterized by the misfolding of Cu/Zn superoxide dismutase (SOD1) into aberrant conformations, which has been pinpointed as a central event in familiar and sporadic forms of the disease (Bosco et al., 2010). *In vitro*, neurons secrete exosomes loaded with mutant SOD1 to the extracellular space (Gomes et al., 2007), where these exosomes are able to transmit the pathogenic trait to other neurons, and propagate the misfolding of SOD1 (Grad et al., 2014). Furthermore, astrocytes also secrete SOD1-loaded exosomes that are selectively toxic for motor neurons (Basso et al., 2013), showing that exosome-mediated transfer of mutant SOD1 is pathogenic.

TDP-43 is a nuclear protein that mislocalizes to the cytoplasm, aggregates and forms inclusions in ALS and in fronto-temporal dementia (FTD). Dipeptide repeat proteins (DPRs) are produced by the unconventional translation of the *C9orf72*, the most common mutated gene in ALS and FTD as well. Recently, both molecules have been associated with exosomal release and transport (Nonaka et al., 2013; Ding et al., 2015; Westergard et al., 2016), further suggesting a link between exosome-mediated communication and the disease.

Exosomes can also propagate toxic molecules in an inter-species manner. An interesting work showed recently a cell-to-cell propagation of mutant Huntingtin (mtHtt) in exosomes using 143 CAG repeats fibroblasts and SH-SY5Y neuroblastoma cells. Furthermore, injecting newborn wild-type mice with human exosomes carrying mtHtt triggered Huntington disease-like (HD) symptoms and pathology in these animals (Jeon et al., 2016), this being the first report of human-to-mouse exosome-mediated propagation of toxic molecules.

### Multiple sclerosis and inflammation

Inflammation is a central player in most neurodegenerative diseases. Neuroinflammatory processes exert profound changes not only in the vicinity but beyond the local environment of cells. In addition to the propagation of toxic proteins, exosomes also carry important inflammatory modulators, such as mRNAs, miRNAs, and cytokines (Gupta and Pulliam, 2014). Microglia, the resident macrophages of the CNS, can shed exosomes loaded with pro-inflammatory molecules such as IL-1β (Bianco et al., 2005) and other cytokines. Their scavenging functions are also crucial in the clearance of toxic seeds such as Aβ (Yuyama et al., 2012). Furthermore, endocrine signals from hematopoietic cells directed to the brain can be transported by exosomes, a phenomenon that is augmented in a context of inflammation (Ridder et al., 2014). It is interesting to note that extracellular vesicles can readily cross the blood brain barrier, adding a communication channel by which systemic inflammation can modulate physiological processes in the CNS.

Multiple sclerosis (MS) is a demyelinating disease characterized by the loss of oligodendrocytes, where inflammation plays a prominent role (Compston and Coles, 2008). Increased levels of circulating exosomes are present in the inflammation-driven disease model of MS, experimental autoimmune encephalomyelitis (EAE). In this model, pro-inflammatory cytokines promote exosome release by immune cells, which in turn carry more pro-inflammatory molecules, spreading inflammation. Animals where exosome-release activity had been hampered showed reduced inflammation and EAE symptoms (Verderio et al., 2012). Another component in the pathogenesis of EAE is glutamate excitotoxicity (Pitt et al., 2000), which is characterized by pathological accumulation of this neurotransmitter and amino acids, among other features. Glutamate can indeed trigger the release of exosomes by oligodendrocytes (Fruhbeis et al., 2013), cells whose homeostasis is known to be altered in demyelinating diseases (Matute et al., 2001). These oligodendrocyte-derived exosomes regulate axon myelination (Bakhti et al., 2011). Nevertheless, despite accumulating evidence suggesting a role of exosomes in inflammatory processes during pathology, whether these extracellular vesicles participate in the interplay between inflammation and excitotoxicity in demyelinating diseases remains unanswered.

## Exosomes as therapeutic and diagnostic tools

### Potential of exosomes as carriers for disease-modifying strategies

**Figure 2:**
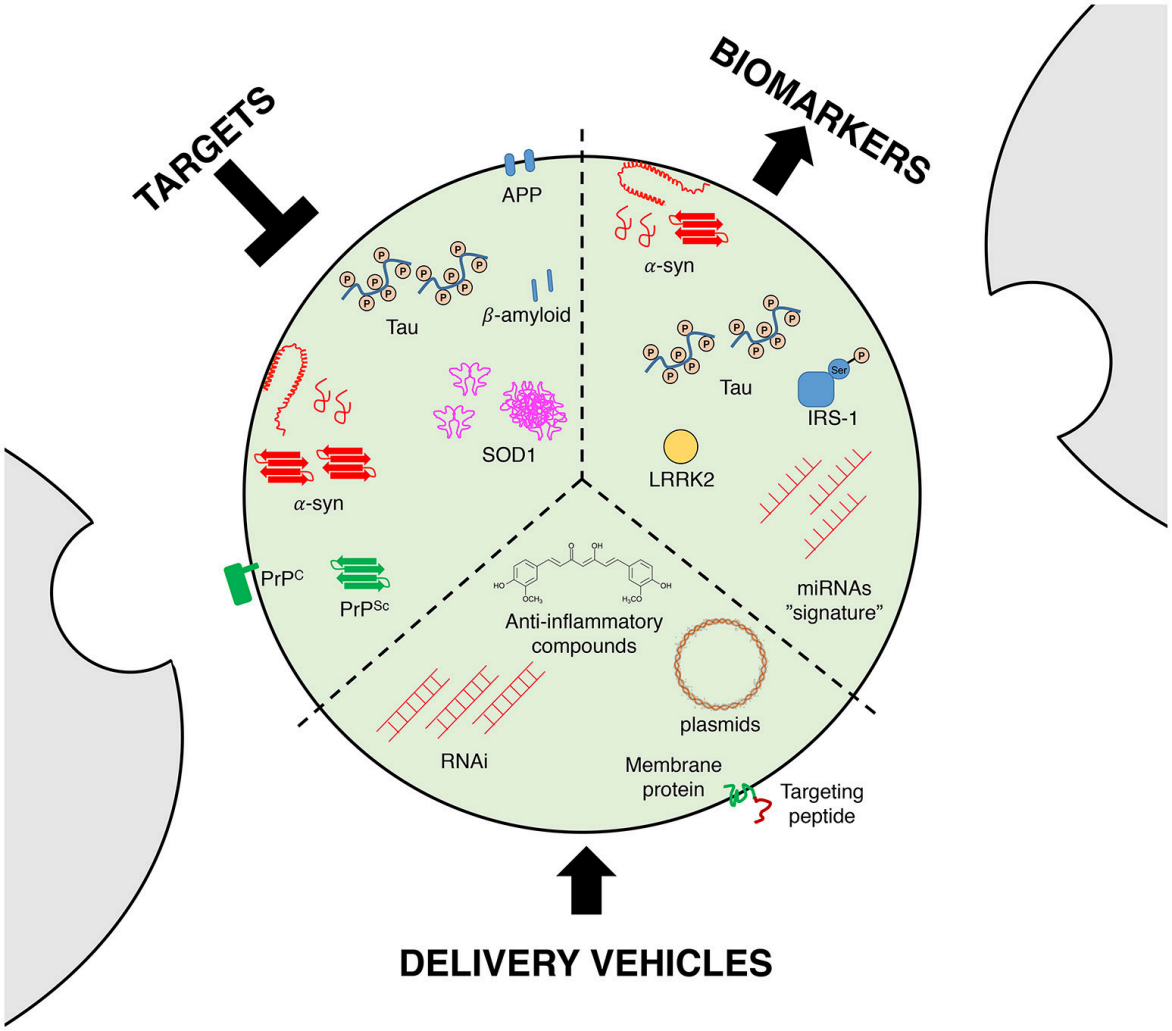
**Exosomes as therapeutic and diagnostic tools**. Targeting the exosomal secretory pathway in neurodegenerative diseases has been proposed as a disease-modifying strategy, since it can reduce the propagation of proteopathic seeds and other toxic molecules. Exosomes can also serve as biomarkers, since they carry molecules relevant for diagnosis, such as disease-related proteins or unique sets of miRNAs (a proposed “signature” or “bar-code”). Finally, the resilience of exosomes in the extracellular milieu renders them as ideal vehicles for the delivery of drugs or disease-modifying molecules such as viral DNA or siRNAs, with the possibility of being targeted to specific cell types or tissues by genetic engineering of membrane proteins.

Although the exact mechanism of exosomal entry into the brain is not fully understood, it should be noted that exosomes are able to cross the blood brain barrier (Record et al., 2011; Tominaga et al., 2015), rendering them as ideal vehicles to delivery molecules into the brain (Figure 2). Exosomes are able to transfer mRNAs, microRNAs or proteins, and they are thought to be an exchange mechanism between cells (Valadi et al., 2007; Simpson et al., 2008), suggesting that these vesicles could be a new delivery machinery especially for RNA interference (RNAi; Valadi et al., 2007; Simons and Raposo, 2009). Indeed, RNAi-based technologies need vectors, since siRNAs can be degraded by extracellular endonucleases present in the serum, cells or extracellular space, and are also immunogenic (Whitehead et al., 2011; Kalani et al., 2014). Regarding delivery, exosomes harbor interesting advantages over other delivery systems such as liposomes, since they have a prolonged half-life and no immunogenicity (Kalani et al., 2014).

The pioneer work of Alvarez-Erviti and coworkers has shown that exosomes can be engineered to target specific tissues (Alvarez-Erviti et al., 2011b). They inserted brain- or muscle-specific peptides in one of the most abundant exosomal membrane proteins: the lysosomal-associated membrane protein 2b (LAMP-2B). In the brain, the proof-of-concept was obtained by using exosomes that contain siRNA for brain-specific knockdown of *BACE1*, encoding the beta-secretase 1 enzyme, in wild type mice (Alvarez-Erviti et al., 2011b). In a more recent study, the same strategy was successfully used to knockdown α-syn in transgenic mouse brain (Cooper et al., 2014). Altogether, these results suggest that exosomes might be interesting vehicles for RNAi delivery. As exosomes are able to target most brain cell types, these results pave the way to applicability in other diseases associated to α-syn accumulation, such as MSA.

microRNAs are highly abundant and largely studied in the biology of exosomes, in part due to their importance in the regulation of gene expression. Therefore, delivery of miRNAs-containing exosomes can constitute a precious therapeutic tool (Ohno et al., 2013). In this sense, MSCs release exosomes that contain overexpressed miRNA-133b, which was found to improve the recovery of ischemic (MCAO) rats, including neurite remodeling in the infarcted area (Xin et al., 2016). The authors used *in vitro* exosomes from MSCs along with overexpression of miR-133b^+^, which increased the release of exosomes from oxygen and glucose-deprived astrocytes. These experiments resulted in increased neurite branching, which suggests that secondary astrocyte release may contribute to neuronal plasticity and recovery after stroke. It should be noted that whereas *in vivo* MSCs are in charge of miRNA-containing exosome release, Xin et al. used astrocytes for the *in vitro* studies, a different cell type where the role and function of miRNAs might be substantially different. Interestingly, five miRNAs from the exosome-released pool showed differential expression levels during traumatic brain injury (TBI), for which miR-21 showed the larger change (Harrison et al., 2016). The authors hypothesize that the increase in miR-21 is due to its release in neuronal exosomes close to the injury boundary, in which vicinity reactive microglia was found.

Engineered nucleic acids, such as hydrophobically modified small interfering RNAs (hsiRNAs) have been shown to load into exosomes (Didiot et al., 2016) and they constitute another example of the therapeutic potential of exosomes. In this work, hsiRNAs targeting Huntingtin (Htt) mRNA were efficiently internalized by primary cortical neurons in a dose-dependent manner, where they silenced up to 35% of Htt mRNA. Lastly, exosomes can also be carriers for neuron-specific rabies viral glycoprotein (RVG) peptide on the membrane, which delivers opioid receptor mu (MOR) siRNA into the brain associated with argonaute 2 (AGO2; Liu et al., 2015).

In addition to nucleic acids, exosomes have been successfully used to deliver drugs (Tian et al., 2014), and exosomes filled with anti-inflammatory compounds such as curcumin have been successfully administered to the brain through an intranasal route (Zhuang et al., 2011). In this experiment, microglia internalized exosomes within 60 min after delivery, and exosome-delivered curcumin successfully attenuated EAE symptoms. Although the distribution was mostly limited to olfactory bulbs, these results suggest that exosomes could be used broadly as molecule carriers for neurological disorders.

### Targeting exosomes as a disease-modifying strategy itself

As mentioned earlier, exosomes are involved in the cell-to-cell transmission of neurodegenerative-related proteins. Indeed, several reports highlight that PrP, tau or α-syn can be released in exosomes (Lee et al., 2005; Aguzzi and Rajendran, 2009; Emmanouilidou et al., 2010; Saman et al., 2012). Taking into account the propagation of α-syn as an example, the total amount of α-syn in exosomes is low under basal conditions (Emmanouilidou et al., 2010). However, in pathological conditions exosomes could allow spreading between cells of pathological misfolded α-syn. This “Trojan-horse” hypothesis leads to hypothesize that targeting exosome release might be a promising strategy to block disease propagation and progression (Figure 2). Future studies are still needed to unravel the relevance of such mechanism, as well as the molecular machinery underlying the import of neurodegenerative-related proteins into exosomes. Furthermore, targeting the exosomal secretory pathway without affecting its physiological communicational role might not be an easy task, and further work onto the basic physiological mechanisms of exosome release and cargo selection will be crucial in this matter.

### Exosomes as biomarkers for disease

Since the identification of neurodegenerative disease related proteins in exosomes, these vesicles have been proposed as putative biomarkers to monitor disease progression (Figure 2). Exosomes can be isolated from multiple fluids including urine, blood or cerebrospinal fluid (CSF), which facilitates their clinical use as biomarkers (Street et al., 2012; Cheng et al., 2014a,b). Quantifications of neurodegenerative disease related proteins in total CSF or in exosomes purified from CSF have led to contradictory results in both AD and PD (Parnetti et al., 2013; Kim et al., 2016; Vella et al., 2016). Interestingly, a report has shown that hyperphosphorylated-tau (often considered as pathological tau) in exosomes is significantly increased in patients with mild forms of AD compared to controls (Saman et al., 2012). Although such result suggests that measuring tau phosphorylation in exosomes could be used as a biomarker for AD, the fact that moderate or severe forms of the disease do not show similar phosphorylation levels dampens that hypothesis. One possible explanation could be that high levels of phosphorylated tau in exosomes is specific of a disease-stage, and not the entire pathological process. Regarding PD, similar divergences are also present in the literature. Indeed, a report showed that α-syn is increased in microvesicles for PD patients while another did not observed variation in the α-syn content (Shi et al., 2014; Tomlinson et al., 2015). This discrepancy could be explained by technical differences as well as differences in the patient population.

As many other proteins are present in exosomes, recent initiatives have used large-scale methodologies to analyze exosome content. Using mass-spectrometry to analyze circulating microvesicles, it was identified that PD patient fibroblasts are enriched in syntenin 1, a regulator of exosome biogenesis (Tomlinson et al., 2015). Similarly, a set of nine miRNAs have been shown to be distinct in exosomes purified from control and prion-infected cells (Bellingham et al., 2012a), defining a molecular “signature” that can identify a pathological process. Although the relevance of such hits in the disease is not established yet, these approaches suggest that establishing a “bar-code” from the exosomal profile of patients might be a more valuable diagnostic tool than quantifying specific disease-related proteins, which are often mixed at early disease stages.

Exosomes can originate from most cell types and have been identified in most body fluids. A study identified the Leucine-Rich Repeat Kinase 2 (LRRK2), a protein involved in some hereditary forms of PD, in urinary exosomes (Fraser et al., 2013). The strong variability observed in clinical populations argued against its use as a biomarker for PD. However, one can hypothesize that exosomes originating from brain might be diluted in body fluids containing exosomes originating from other organs. To test this hypothesis, a recent study used an immunochemical method to purify brain exosomes from peripheral blood plasma (Shi et al., 2014). The authors reported an increase of α-syn specifically in exosomes from plasma of PD patients (i.e., no overall modification was observed in total plasma). Although this approach was developed to be brain-specific based on the expression of the L1-cell adhesion molecule (L1-CAM), this protein is also expressed in the renal system (Allory et al., 2008; Vella et al., 2016), therefore these results have to be taken carefully. However, and more importantly, the immunochemical capture method provides an innovative approach for quantifying neurodegenerative disease related proteins in peripheral fluids to be used as biomarkers, and which hold the promise of a better specificity (Vella et al., 2016).

Interestingly, insulin resistance (i.e., decreased insulin/insulin like growth factor IGF-1 signaling) has been established in both MSA patients and in a well-characterized transgenic model, the PLP-SYN mice (Bassil et al., 2017). Insulin resistance can be measured by analyzing the amount of the downstream messenger insulin receptor substrate-1 phosphorylated at serine residues 312 (IRS-1pS312) or 616 (IRS-1pS616; Moloney et al., 2010; Talbot et al., 2012). Since this biomarker can be traced in peripheral exosomes (Kapogiannis et al., 2015), we evaluated the murine plasma neural derived exosomal IRS-1pS307 levels (corresponding to human IRS-1pS312) in transgenic MSA mice that were treated with the glucagon-like peptide-1 (GLP-1) agonist exenatide. We found a correlation with the number of nigral dopaminergic neurons and with striatal oligomeric α-syn load in transgenic MSA mice, indicating that PLP-SYN mice with highest plasma exosomal IRS-1pS307 concentrations had a lower number of nigral TH and Nissl-stained neurons as well as higher striatal oligomeric α-syn levels (Bassil et al., 2017). Altogether, these data suggest that peripheral neural derived exosomal IRS-1pS312 is an exosomal biomarker candidate that may serve as an objective outcome measure of target engagement for preclinical studies as well as clinical trials with GLP-1 analogs and other compounds modulating insulin/IGF-1 signaling.

## Concluding remarks

The last decade has witnessed a dramatic increase in publications in the field of exosomes and related extracellular vesicles. Initially considered waste disposal material, recent evidence has progressively changed this view, and exosomes are currently considered, in fact, as a broad intercellular communication system that can internalize, transport and transfer all types of biomolecules, from nucleic acids to peptides and proteins.

Most of the research in exosomes and extracellular vesicles has been carried out in cell cultures. This *in vitro* approach has been very useful to screen cell types, cargos, and genetic backgrounds, providing useful data to understand the biogenesis and physiological role of exosomes. However, their role in neurodegenerative diseases, either as carriers of disease-modifying molecules, or as an effective clearance system, is yet to be completely understood. Several questions remain unanswered, mostly because of the lack of *in vivo* studies, as well as experiments in more intact preparations where the relevant molecules are expressed in endogenous levels. Among several open questions in the field, of particular interest, are (i) whether exosomes from diseased organisms can effectively propagate the pathogenic trait *in vivo*, (ii) whether targeting the exosomal secretory pathway can be beneficial for the pathology, and (iii) what is the molecular profile or “signature” that can be read from exosomes isolated from patients to be used as biomarkers in different brain disorders. Future studies will hopefully fulfill the high expectations raised among neurobiologists regarding the use of exosomes as therapeutic targets and/or tools.

## Author contributions

FS, OP, MB, and BD were responsible for the conception of the article. FS, OP, MB, and BD did the scientific literature review. FS created the figures. FS, OP, MB, and BD wrote the first draft of the article. FS, OP, MB, WM, EB, and BD critically revised the entire manuscript and approved the final version.

### Conflict of interest statement

The authors declare that the research was conducted in the absence of any commercial or financial relationships that could be construed as a potential conflict of interest.
